# A Scalable Organoid Model of Urothelial Aging for Metabolic Interrogation, Infection Modeling, and Reversal of Age‐Associated Changes

**DOI:** 10.1111/acel.70391

**Published:** 2026-01-23

**Authors:** Adwaita R. Parab, Arnold M. Salazar, Steven J. Bark, Margarita Divenko, Vasanta Putluri, D'Feau J. Lieu, Aadya S. Singh, Nagireddy Putluri, Indira U. Mysorekar

**Affiliations:** ^1^ Department of Medicine, Section of Infectious Diseases Baylor College of Medicine Houston Texas USA; ^2^ Department of Medicine, Section of Gastroenterology Baylor College of Medicine Houston Texas USA; ^3^ Advanced Technology Cores Baylor College of Medicine Houston Texas USA; ^4^ Department of Molecular and Cellular Biology Baylor College of Medicine Houston Texas USA; ^5^ Department of Molecular Virology and Microbiology Baylor College of Medicine Houston Texas USA; ^6^ Huffington Center of Aging Baylor College of Medicine Houston Texas USA

**Keywords:** assembloids, bladder, d‐mannose, *Irg1*, metabolomics, UPEC, urothelium, UTI

## Abstract

Aging leads to a progressive decline in overall bladder function resulting in lower urinary tract symptoms and increased susceptibility to infections. However, tissue‐specific mechanisms of aging, specifically the contributions of the urothelium, remain elusive. Here, we introduce mouse bladder epithelium‐derived organoids (mBEDOs) as a scalable platform to model urothelial aging. mBEDOs from aged mice recapitulate key features of age‐associated cellular reprogramming, including oxidative stress, senescence, and DNA damage. We demonstrate the utility of mBEDOs for modeling Uropathogenic *Escherichia coli* (UPEC) infection, generating assembloids between mBEDOs and macrophages to model epithelial‐immune interactions, and genetic perturbation. Using the mBEDO platform, we also identify urothelium‐specific changes in purine, amino acid, and glycerophospholipid metabolism, which may contribute to age‐associated cellular perturbations. Lastly, supplementation with depleted metabolites, nicotinamide and d‐mannose, reduces DNA damage and oxidative stress and restores mitochondrial integrity in aged mBEDOs. These findings establish mBEDOs as an effective platform for investigating molecular and cellular underpinnings of urothelial aging and exploring metabolism‐based interventions for age‐associated bladder dysfunction.

## Graphical abstract

mBEDOs: a scalable platform for modeling urothelial aging. mBEDOs derived from aged mouse bladders capture essential molecular and cellular features of urothelial aging and provide a versatile system for high‐throughput metabolic profiling, therapeutic screening via metabolite supplementation, age‐ and sex‐based analyses, infection modeling, and immune–epithelial interaction studies.

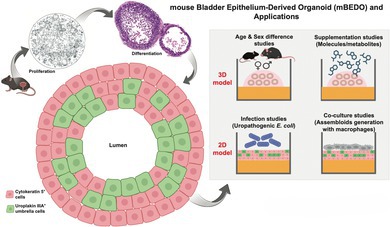

## 1 Introduction

Aging is a principal driver of disease, characterized by diminished cellular homeostasis, impaired tissue repair, and increased susceptibility to infection (López‐Otín et al. 2013). Aging is detrimental in epithelial barrier tissues, which serve as the first line of defense against environmental insults. In the lower urinary tract, age‐associated effects include reduced bladder capacity, overactive bladder, decreased urethral pressure, and higher levels of residual urine in the bladder (Nishii 2021). These functional issues manifest in the form of lower urinary tract symptoms (LUTS) such as urinary incontinence, lack of voiding control, bladder pain syndrome, and recurrent urinary tract infections (rUTIs) (Hu et al. 2004).

The bladder epithelium (urothelium) protects against toxins and pathogens and responds to mechanical stressors such as stretch and osmotic pressure (Jafari and Rohn 2022). We reported that aged female mice exhibit hallmarks of urothelial aging, including increased senescence, spontaneous pyroptosis, oxidative stress, mitochondrial dysfunction, and impaired autophagy with lysosomal accumulation, which heighten UTI susceptibility (Joshi et al. 2024). In parallel, aging also affects bladder mucosal immunity (Ligon et al. 2023). Bladders, like other mucosal tissues, also contain a diverse population of resident immune cells, with macrophages, the most abundant subset, forming a dense network beneath the urothelium, regulating tissue homeostasis and epithelial barrier maintenance (Lacerda Mariano and Ingersoll 2018, 2020). However, with age, B and T cells and macrophages organize into bladder tertiary lymphoid tissue (bTLT) (Ligon et al. 2020). In the bladder, examining urothelium‐specific aging phenotypes in vivo is complicated by interactions with immune cells, vasculature, neurons, and smooth muscle. This highlights the need for model systems for epithelial‐intrinsic changes in isolation while also enabling study of epithelial‐immune interactions.

Human and rodent models have advanced our understanding of bladder aging. However, studying the functional consequences of aging in a tissue‐specific manner, particularly through genetic perturbation, remains a significant challenge. Performing loss‐of‐function studies in aged mice is time‐intensive, costly, and often not feasible at scale, requiring aging colonies over many months and coordinating treatment windows late in life (McLean et al. 1992). Thus, complementary models that retain the strengths of mouse biology while enabling faster, more tractable interrogation of aging mechanisms in the urothelium are urgently needed.

The earliest studies describing ex vivo urothelial cell cultures date back to the 2000s when ex vivo urothelial constructs expressing uroplakins and cytokeratin 20 and retaining barrier characteristics were developed (Varley and Southgate 2011; Vasyutin et al. 2019). Human urothelial cell monolayers with a stratified urothelium were generated from human bladder washings (Nagele et al. 2008). Recently, a urine‐tolerant urothelial organoid model derived from commercially available human progenitors successfully modeled infection (Horsley et al. 2018; Smith et al. 2006). Another study showed interaction between the immune and epithelial compartments during infection, using a 3D mouse urothelial organoid model with bacterial invasion by UPEC and neutrophil response in an ex vivo system (Sharma et al. 2021). Recently, a report showed the applicability of a human bladder organoid for modeling UTIs (Zulk et al. 2025). Additionally, studies showed urothelial organoids derived from both mouse and human tissue to study bladder cancer (Kim et al. 2020; Lee et al. 2018; Minoli et al. 2023; Mullenders et al. 2019; Sharma et al. 2021; Shin et al. 2011; Viergever et al. 2024; Yoshida et al. 2018) and the use of bladder assembloids with stromal components and muscle layer to recapitulate the in vivo tumor microenvironment (Kim et al. 2020). However, there is a lack of organoid models that explicitly capture aging‐associated molecular, cellular, and metabolic alterations.

In this report, we describe the development of aged mouse bladder epithelium‐derived organoids (mBEDOs) as a scalable, tissue‐specific model of urothelial aging. We demonstrate that mBEDOs from aged mice recapitulate key features of in vivo urothelial aging, including elevated oxidative stress, DNA damage, and cellular senescence. mBEDOs can be cultured in both 2D and 3D formats and extended into assembloids, incorporating immune cells, enabling modeling of uropathogenic *Escherichia coli* (UPEC) infection and epithelial–immune interactions. Importantly, mBEDOs can be generated from genetically modified mice, offering a unique platform to examine the role of specific genes like the immune response gene 1 (*Irg1*) in the context of epithelial aging. Metabolomic characterization of young and aged mBEDOs and those generated from *Irg1* ^*−/−*^ mice using untargeted LC–MS/MS metabolomics identified distinct metabolic signatures of age. Finally, supplementing aged mBEDOs with d‐mannose and nicotinamide (NAM), two metabolites depleted in aged samples, significantly reduced oxidative stress and DNA damage while restoring mitochondrial integrity. Together, these findings position mBEDOs and assembloids as powerful tools for dissecting epithelial aging, modeling infection susceptibility, and evaluating metabolism‐based interventions in a high‐throughput, tissue‐specific context.

## 2 Methods

### 2.1 Mice

Wild‐type (WT) young (2–3 months old) and aged (15–18 months old) C57BL/6J mice were obtained from the National Institute on Aging (NIA). *Irg1* ^*−/−*^ mice (C57BL/6NJ‐Acod1em1(IMPC)J/J) were originally obtained from the Jackson Laboratory and maintained in our animal facility. Mice were held in a pathogen‐free and temperature‐controlled facility with a 12‐h light/dark photocycle. All procedures were performed in accordance with institutional guidelines and were approved by the Institutional Animal Care and Use Committee at Baylor College of Medicine (Protocol No. AN‐8629).

### 2.2 Mouse Bladder Organoid Generation

Young and aged female C57BL/6 mice (*n* = 20 per group) were sacrificed, and bladders were harvested, rinsed in ice‐cold 1× PBS, and minced with a scalpel. Tissue from individual mice was digested in 1 mL Advanced DMEM containing 5 mg/mL Collagenase II, 10 mg/mL elastase (20 μL), and 10 mM Y‐27632 (0.5 μL), with rotation at 37°C for 4 h (Roto‐Therm‐Mini Mix Plus, 10 rpm). Digests were centrifuged (1500 RCF, 6 min, 4°C), supernatants removed, and pellets incubated in 1 mL pre‐warmed TrypLE for 20 min at 37°C with rotation. Cells were washed, resuspended in Advanced DMEM supplemented with 1.25% GlutaMAX, counted using an Invitrogen Countess 3 automated cell counter, and embedded at 20,000 cells per 50 μL Matrigel. Up to five Matrigel domes were plated per well in pre‐warmed 6‐well suspension plates and polymerized at 37°C for 15 min.

Organoids were cultured in proliferation medium consisting of Advanced DMEM supplemented with 1% B27, 0.25% N‐acetyl‐l‐cysteine, 0.5% nicotinamide, 0.01% mouse EGF, 0.04% A‐83‐01, 2% Noggin, 10% R‐spondin (Noggin and R‐spondin from the Digestive Diseases Core, Baylor College of Medicine), 1.25% GlutaMAX, and 1% Penicillin–Streptomycin. Medium was refreshed every 2 days. After 7 days, organoids were transitioned to intermediate medium consisting of proliferation medium supplemented with 12% WNT‐3A and 10 pg/mL mouse IL‐6, which was maintained from days 7–14 with medium changes every 2 days. On day 14, organoids were transferred to differentiation medium containing Advanced DMEM supplemented with 20% FBS, 1% B27, 0.04% A‐83‐01, 25 ng/mL mouse FGF‐7, and 100 ng/mL mouse FGF‐10 for 7 days. Mature organoids could subsequently be maintained in Advanced DMEM containing 10% FBS and Penicillin–Streptomycin for approximately 10 days. Reagent catalog numbers available in Table S1.

### 2.3 Organoid Passaging and Cryopreservation

For passaging, organoids were subcultured at the end of the proliferation stage and could be maintained for up to 10 passages. Matrigel domes were mechanically disrupted by gentle scraping and transferred to 15 mL tubes. Organoids were pelleted (1500 RCF, 6 min, 4°C), washed three times with ice‐cold 1× PBS, and resuspended in pre‐warmed Advanced DMEM. Cells were counted and replated at 20,000 cells per 50 μL Matrigel in pre‐warmed 6‐well plates as described above. For cryopreservation, organoids were resuspended at 1 × 10^6^ cells/mL in Recovery Cell Culture Freezing Medium and stored in liquid nitrogen.

### 2.4 Organoid Preparation for Immunohistochemistry and Histology

Organoids cultured in 6‐well plates were placed on ice for 10 min, after which Matrigel domes were gently disrupted using a diagonally cut 1000‐μL pipette tip and scraped from the well bottom. Organoid suspensions were transferred to 15‐mL conical tubes, incubated on ice for an additional 10 min, and centrifuged at 1500 RCF for 5 min at 4°C. Supernatants were discarded, and pellets were washed three times with ice‐cold 1× PBS, centrifuging between washes under the same conditions. Following the final wash, organoids were resuspended in 1 mL 1× PBS, transferred to 1.5‐mL tubes, and pelleted again. Organoid pellets were embedded in OCT by layering a thin suspension of pelleted organoids into embedding cassettes, frozen at −20°C for 1 h, and stored at −80°C. Cryosections (10 μm) were prepared using a cryostat and stored at −80°C until use.

### 2.5 Immunofluorescence Staining of Organoid Sections

Formalin‐fixed paraffin‐embedded organoid sections from young and aged mice were deparaffinized in three changes of 100% Histo‐Clear (5 min each) and rehydrated through a graded ethanol series (100%, 90%, 70%, and 50%; 5 min each), followed by rinsing in 1× PBS. Sections were blocked with 1% bovine serum albumin (BSA) in 1× PBS for 1 h at room temperature. Primary antibodies—anti‐UpkIIIA (1:1000), anti‐Ck5 (1:500), anti‐γH2AX (1:500), or anti–SA‐β‐Gal (1:500)—were diluted in 1% BSA with 0.1% Tween‐20 and applied overnight at 4°C. Slides were washed three times in 1× PBS (5 min each) and incubated with appropriate secondary antibodies (1:1000) for 1 h at room temperature, followed by three additional PBS washes. Sections were mounted with ProLong Gold Antifade reagent containing DAPI (Invitrogen, P36931), coverslipped, and sealed with clear nail polish. Images were acquired using a Nikon Ti2 ECLIPSE confocal microscope (40× objective, NA 1.4). Fluorescence intensity was quantified from 5 independent mBEDO batches, with five regions of interest (ROIs) analyzed per sample using Nikon NIS‐Elements software. Mean fluorescence intensity for each target was quantified per ROI and normalized to the corresponding DAPI fluorescence to account for differences in cell number, nuclear density, and organoid thickness between conditions.

### 2.6 Immunofluorescence Staining of 3D mBEDOs in Suspension

For whole‐mount immunofluorescence, mBEDOs embedded in Matrigel were gently released using a diagonally cut P1000 pipette tip and transferred to 15‐mL tubes. Samples were incubated on ice for 15 min, centrifuged (1500 RCF, 5 min, 4°C), and washed once with ice‐cold 1× PBS, followed by a second incubation on ice and centrifugation. Organoids were transferred to 1.5‐mL tubes and blocked/permeabilized overnight at 4°C in 1× PBS containing 5% FBS, 4% BSA, and 0.5% Triton X‐100.

Primary antibody staining (anti‐Ck5) was performed overnight at 4°C in 1× PBS containing 5% FBS, 4% BSA, and 0.1% Tween‐20. Organoids were washed three times with 1× PBS containing 0.01% BSA, centrifuging gently between washes, and incubated with Goat anti‐Rabbit Alexa Fluor 594 secondary antibody (1:1000) for 1 h at room temperature. Following three additional washes, organoids were mounted in suspension using silicone isolators (13 mm diameter × 0.8 mm depth) and immersed in glycerol clearing solution (prepared by mixing 110 mL glycerol, 23.3 mL water, and 100 g fructose to a final volume of 220 mL). Images were acquired using a Nikon Ti2 ECLIPSE confocal microscope (40× objective, NA 1.4).

### 2.7 Hematoxylin and Eosin (H&E) Staining of mBEDOs

Frozen OCT‐embedded mBEDO sections were fixed in 4% paraformaldehyde for 10 min, rinsed three times in 1× PBS (3 min each), and rehydrated through a graded ethanol series (100%, 95%, 70%, and 50%; 5 min each). Sections were rinsed in distilled water to remove residual alcohol and stained with hematoxylin for 30 s. Excess stain was removed under running distilled water, followed by brief differentiation in acid alcohol (1% HCl in 70% ethanol) and bluing in sodium bicarbonate solution for 3 min. Sections were dehydrated in 90% ethanol, counterstained with eosin (five dips), and further dehydrated through increasing ethanol concentrations (50%, 70%, 95%, and 100%; 5 min each). Slides were cleared in Histo‐Clear for 2 min and mounted with Permount mounting medium and coverslips. Slides were cured overnight, sealed with nail polish, and imaged using a Panoramic MIDI slide scanner (3DHISTECH Ltd., Hungary).

### 2.8 RNA Isolation and RT–qPCR Analysis

Each frozen pellet corresponding to a single Matrigel dome containing organoids was reserved for RNA extraction and stored at −80°C in 1.5‐mL tubes containing 500 μL TRIzol. Total RNA was isolated from frozen pellets derived from young and aged mBEDOs. Samples were thawed on ice, mixed with 100 μL chloroform, inverted vigorously until cloudy, incubated on ice for 10 min, and centrifuged at 12,000 RCF for 15 min at 4°C to achieve phase separation. The aqueous (RNA) phase was carefully transferred to a fresh tube, and RNA was precipitated by addition of 250 μL isopropanol, followed by inversion and incubation on ice for 10 min. Samples were centrifuged at 12,000 RCF for 15 min at 4°C, supernatants discarded, and RNA pellets washed with 1 mL 75% ethanol. After centrifugation under the same conditions, pellets were air‐dried at room temperature for 15 min and resuspended in 40 μL molecular biology–grade water. RNA was treated with DNase I according to the manufacturer's instructions.

cDNA was synthesized from 100 ng total RNA using SuperScript II Reverse Transcriptase, and all cDNA samples were diluted 1:8 with RNase‐free water. RT‐qPCR reactions were prepared using SsoAdvanced Universal SYBR Green Supermix in 10‐μL reactions (5 μL SYBR mix, 1 μL each forward and reverse primer, 2 μL diluted cDNA, and 1 μL RNase‐free water). Primer sequences are listed in Table S2. Reactions were run in triplicate with GAPDH as the reference gene on a QuantStudio 3 Real‐Time PCR System (Applied Biosystems) using the following cycling conditions: 98°C for 3 min, followed by 40 cycles of 98°C for 30 s and 58°C for 30 s. Raw Ct values were used to calculate relative fold changes normalized to young mBEDO samples. Data visualization and statistical analyses were performed using GraphPad Prism.

### 2.9 Derivation of 2D Monolayers From 3D Organoids

3D organoids were released from Matrigel, pelleted (1500 RCF, 5 min, 4°C), dissociated with TrypLE (20 min, 37°C), and resuspended in Advanced DMEM. Single cells were counted and resuspended in proliferation medium containing 1:20 Matrigel. Cells (100,000/well) were plated onto 24‐well glass‐bottom plates and cultured with daily medium changes for 4 days to generate confluent monolayers.

### 2.10 UPEC Infection of 2D Monolayers

GFP‐expressing UTI89 was grown statically in LB at 37°C for 17–18 h, pelleted, and prepared for infection as described previously (Joshi et al. 2021). Monolayers were infected for 1 or 3 h, washed with 1× PBS, and incubated in medium containing gentamicin (1:500 dilution from 10 mg/mL stock) until 24 h post‐infection. Cells were fixed in 4% PFA (30 min), blocked with 1% BSA, and stained with anti‐UpkIIIA (1:1000) overnight at 4°C, followed by secondary antibody incubation and DAPI counterstaining. Imaging was performed using a Nikon Ti2 ECLIPSE confocal microscope (40×, NA 1.4). Infected monolayers were harvested by scraping, lysed with 0.1% Triton X‐100, and processed for CFU enumeration as previously described in (Joshi et al. 2021). Serial dilutions were plated on LB agar; colonies were averaged from five technical replicates per sample, log_10_‐transformed, and analyzed using GraphPad Prism.

### 2.11 Western Blotting (WB)

mBEDOs were lysed in RIPA buffer (ThermoScientific, 89901) and stored at −80°C. Lysates were sonicated, clarified, and protein concentration determined by BCA assay. Equal amounts of protein (10 μg) were resolved on 4%–20% TGX precast gels (Bio‐Rad) and transferred to PVDF membranes. Membranes were blocked with Intercept (TBS) Blocking Buffer (LI‐COR) and incubated overnight at 4°C with primary antibodies against STING or phospho‐STING (1:1000), with β‐actin as a loading control. Following incubation with appropriate secondary antibodies, signals were detected using a ChemiDoc MP imaging system (Bio‐Rad) and quantified with Image Lab software.

### 2.12 Lysosomal, Mitochondrial and Dihydroethidium Staining

Fresh‐frozen 10‐μm sections of young and aged mBEDOs were stained with LysoTracker Red (75 nM in Advanced DMEM) for lysosomal labeling, MitoTracker Orange CMTMRos and MitoTracker Green FM for mitochondrial labeling, and dihydroethidium (DHE) for reactive oxygen species detection. Sections were incubated with LysoTracker or MitoTracker dyes for 30 min at 37°C, or with DHE (10 mM) for 10 min at 37°C, protected from light. Slides were rinsed with 1× PBS, counterstained with DAPI, coverslipped, and sealed with nail polish. Images were acquired using a Nikon Ti2 ECLIPSE confocal microscope (40×, NA 1.4) and analyzed using NIS‐Elements. Fluorescence intensity for lysosomal, mitochondrial, and DHE staining was quantified from 3 to 5 independent young, aged, treated, and untreated mBEDO samples, with five regions of interest averaged per sample using ImageJ.

### 2.13 BMDM–Organoid Co‐Culture

Bone marrow–derived macrophages (BMDMs) were generated from mouse femurs and tibias as previously described (Symington et al. 2015). Briefly, marrow was flushed, filtered, subjected to RBC lysis, and cultured (1–2 × 10^6^ cells/10‐cm dish) in DMEM/F12 supplemented with 20% FBS, 50 ng/mL M‐CSF, and penicillin/streptomycin. After 7 days, macrophage differentiation was confirmed by flow cytometry (CD11b^+^F4/80^+^). BMDM–organoid co‐culture was adapted from (Noel et al. 2017) with modifications. BMDMs (0.2–2 × 10^6^) were plated onto inverted 0.4‐μm PET transwell inserts and incubated for 2 h at 37°C, then returned upright. Differentiated bladder organoids (100,000 cells) were seeded on the apical surface as monolayers in differentiation medium supplemented with 50 ng/mL M‐CSF and co‐cultured overnight. For whole‐mount immunofluorescence, inserts were fixed with 4% methanol‐free PFA, blocked with 1% BSA/0.1% Triton X‐100, and stained with anti‐Ck5 (1:200) and anti‐F4/80 (1:300) overnight at 4°C, followed by fluorescent secondary antibodies and DAPI mounting. Images were acquired using a Nikon Ti2 ECLIPSE confocal microscope (40×, NA 1.4) and analyzed with NIS‐Elements software.

### 2.14 Metabolite Supplementation of mBEDOs

Fully differentiated, mature young and aged 3D mBEDOs were supplemented with 17.5 mM d‐Mannose or 1 mM nicotinamide (NAM) in Advanced DMEM, 10% FBS, Pen/Strep and GlutaMAX. Organoids were harvested at 24 h post‐treatment with each metabolite. Individual Matrigel tabs were collected at the 24‐h timepoint by gently disrupting the Matrigel tabs and extracting organoids. Matrigel was removed by thawing tubes on ice for 15 min, followed by centrifugation at 1500 RCF at 4°C for 15 min, and removing the supernatant which contained the Matrigel. This led to organoid pellets that were either frozen in OCT (Section 2.4) to perform histological analysis and staining or pelleted and stored in TriZOL for RNA extraction, cDNA synthesis, and qRT‐PCR (Section 2.8).

### 2.15 Targeted Metabolomics of Whole Mouse Bladders

Targeted metabolomics was performed on freshly isolated mouse bladder tissues using established liquid–liquid extraction protocols (Gohlke et al. 2019; Putluri et al. 2011; Vantaku et al. 2019). Samples were homogenized in methanol: water containing an isotopically labeled internal standard mix. Mouse liver and pooled sample extracts were used as quality controls. Metabolites were analyzed by LC–MS/MS using multiple reaction monitoring (MRM) on an Agilent 6495 Triple Quadrupole mass spectrometer coupled to HPLC, with data acquisition and peak integration performed using Agilent MassHunter software. Peak areas were log_2_‐transformed and normalized to internal standards. Differential metabolites were identified using Student's *t*‐test with Benjamini–Hochberg false discovery rate correction (FDR < 0.25).

### 2.16 Untargeted Metabolomics of mBEDOs

Untargeted metabolomics was performed on mBEDOs extracted from Matrigel and homogenized in methanol: water, proteins precipitated with methanol/acetonitrile, dried, and reconstituted in methanol: water. A pooled quality control sample was included throughout analysis. Metabolite separation was performed by reversed‐phase and HILIC chromatography using a Vanquish Horizon UHPLC system with Waters ACQUITY HSS T3 and BEH Amide columns. Data were acquired on an Orbitrap IQ‐X Tribrid mass spectrometer (Thermo Fisher Scientific) operated in positive and negative ion modes. Raw data were processed using Compound Discoverer (v3.3) for peak detection, alignment, and annotation using mzCloud, NIST 2020, HMDB, and in‐house libraries. Peak areas were log_2_‐transformed, median‐IQR normalized, and differential metabolites were identified using unadjusted *p*‐values (*p* < 0.05), as FDR correction across the complete feature list (which includes non‐metabolic/environmental features inherent to the acquisition protocol) markedly reduced statistical power for biologically relevant metabolites.

### 2.17 Quantification and Statistical Analysis

**FIGURE 1:**
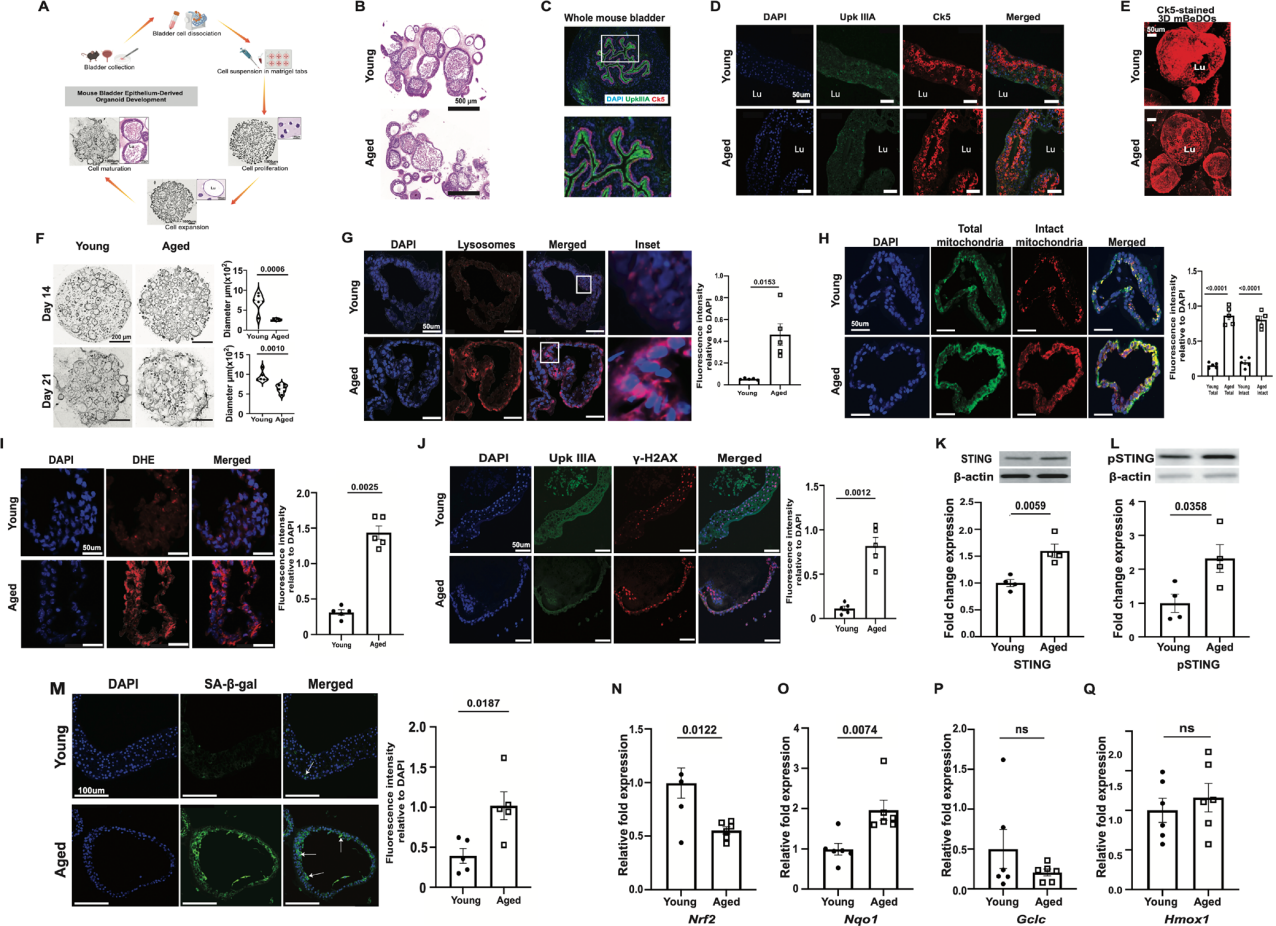
mBEDOs recapitulate multiple key features of in vivo bladders. (A) Schematic diagram of mBEDO generation. (B) H&E staining shows multilayer cellular architecture of differentiated mBEDOs. (C) UpkIIIA and Ck5 expressions in in vivo whole bladder. (D) UpkIIIA and Ck5 expressions in mBEDOs; lumen (Lu). (E) 3D mBEDOs showing Ck5 expression. (F) Brightfield images of mBEDOs in Matrigel, growth quantitation at days 14 and 21 (G) Images and quantitation of lysosome staining (H) Images and quantitation of mitochondrial staining. (I) Images and quantitation of DHE staining (Data presented as mean ± SEM, *n* = 5) (J) Images and quantitation of UpkIIIA and γH2AX staining (Data presented as mean ± SEM, *n* = 5). (K, L) WB quantitation of STING and pSTING (Data presented as mean ± SEM, *n* = 4). (M) Images and quantitation of SA‐β‐gal staining (Data presented as mean ± SEM, *n* = 5). (N–Q) RT‐PCR analyses of *Nrf2, Nqo1, Gclc, Hmox1* (Data presented as mean ± SEM, *n* = 6). All *p* values by two‐tailed unpaired *t*‐test.

**FIGURE 2:**
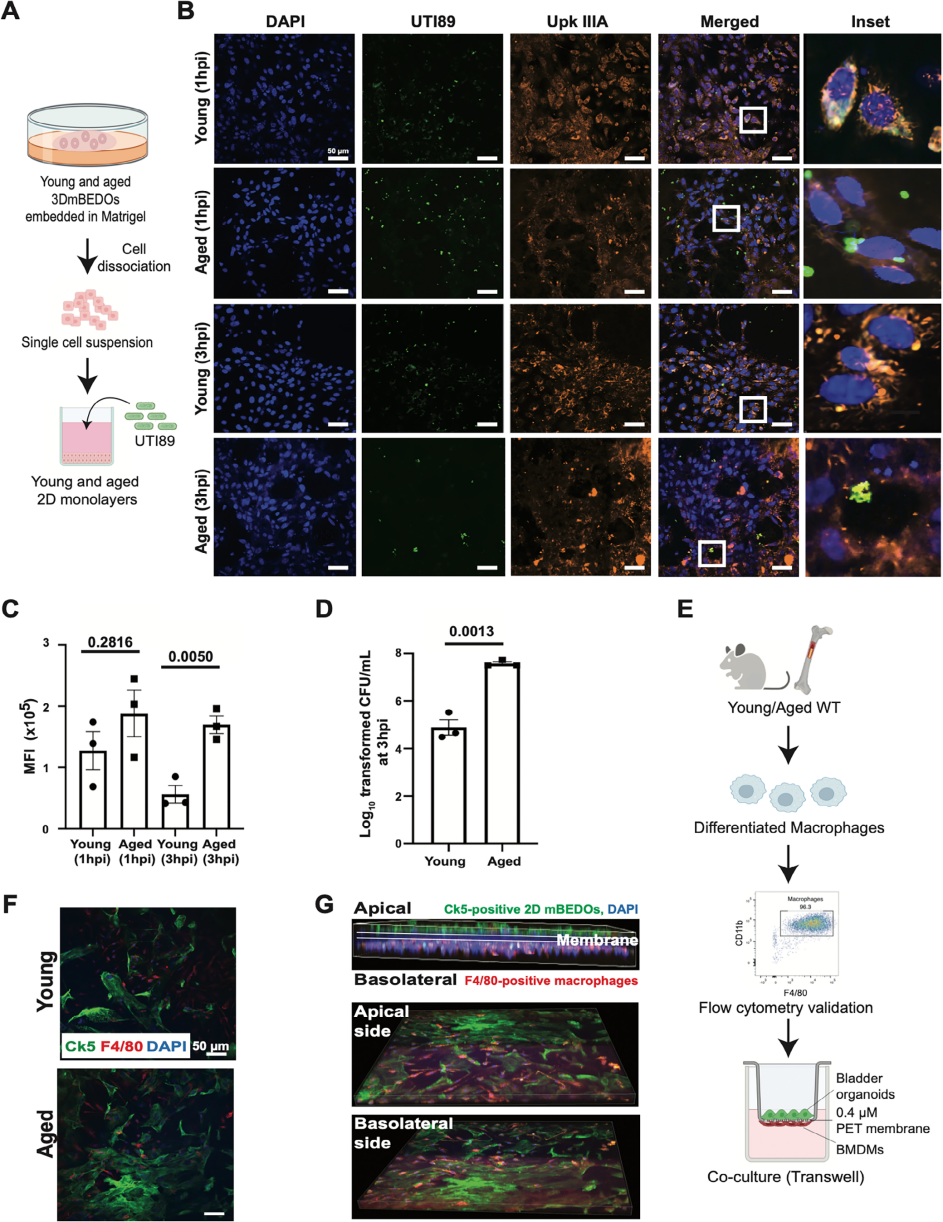
mBEDO platform demonstrates practical utility for UPEC infection and macrophage interaction assays. (A) Schematic diagram of UPEC infection in 2D monolayers. (B) Localizations of UTI89 (green) and UpkIIIA (orange) in 2D monolayers. DAPI (blue) stains nuclei. (C) Quantitation of UTI89 levels at 1 and 3 hpi (Data presented as mean ± SEM, *n* = 3, *p* value by two‐tailed unpaired *t*‐test). (D) CFU quantitation at 3 hpi (Data presented as mean ± SEM, *n* = 3, *p* value by two‐tailed unpaired *t*‐test). (E) Schematic diagram of macrophage co‐culture in 2D monolayers (F) Interaction of macrophages (F4/80, red) and Ck5^+^ cells (green). DAPI (blue) stains nuclei. (G) Representative images of macrophages (F4/80, red) on the basolateral side of transwell inserts.

Sample sizes (biological replicates) were as follows: fluorescence intensity analyses used *n* = 5 for Figure 1 and *n* = 3 for Figures 2 and 5; Western blot analyses used *n* = 4; and qRT‐PCR analyses used *n* = 6 (Figure 1). Metabolomic analyses included *n* = 4 young whole bladders, *n* = 5 aged whole bladders, *n* = 3 young mBEDOs, *n* = 3 aged mBEDOs, and *n* = 3 *Irg1* ^*−/−*^ mBEDOs. Each *n* represents an independent biological replicate (mBEDOs derived from a single mouse and cultured in a single well). All data were analyzed using GraphPad Prism (versions 9.0.1–10.2.2). Two‐tailed unpaired *t*‐tests were used for comparisons between two groups when data approximated a normal distribution. Exact *n* values and statistical tests are indicated in the figure legends. Data are presented as mean ± SEM.

## 3 Results

### 3.1 mBEDOs Recapitulate Key Features of In Vivo Bladders

We generated mouse bladder epithelial organoids (mBEDOs) from whole bladders of young and aged female C57BL/6J mice. Organoids were established through a three‐stage culture system comprising proliferation, maturation, and differentiation phases (Figure 1A). During the initial 7‐day proliferation phase, cells expanded and formed spheroid structures in response to EGF, Noggin, and R‐spondin. Transition to an intermediate medium containing WNT3A and IL‐6 promoted cellular maturation and reduced proliferation, resulting in increased spheroid size. Subsequent differentiation in FBS‐, FGF‐7‐, and FGF‐10–containing medium supported terminal differentiation. By day 21, mBEDOs formed mature, multilayered structures with a central lumen, closely resembling the architecture of the urothelium in vivo (Figure 1A). Organoids were subcultured at the end of the proliferation stage to enable scalability and technical replication, with cell density maintained throughout differentiation. Cells were also cryopreserved to support longitudinal and comparative studies.

H&E staining revealed that differentiated mBEDOs closely resemble the urothelium in vivo (Figure 1B–C). Both basal (Ck5) and apical (UpkIIIA) markers were detected across multiple layers (Figure 1D), with 3D imaging confirming Ck5 enrichment in the outermost layers (Figure 1E). Quantification of organoid growth demonstrated that aged mBEDOs exhibited significantly smaller diameters than young counterparts during both the proliferation (day 14) and differentiation (day 21) stages (Figure 1F), consistent with the reduced growth capacity observed in aged tissues in vivo (Carlson and Conboy 2007) and further adds a feature of in vivo aged urothelium recapitulated in mBEDOs.

Our previous work demonstrated that aged urothelium in vivo exhibits cellular and molecular alterations, including abundant and dysfunctional lysosomes and mitochondria, increased reactive oxygen species (ROS), reduced antioxidant responses, and a senescence‐associated phenotype (Joshi et al. 2024). To assess whether these features are recapitulated in mBEDOs, fresh‐frozen sections were stained for lysosomes and quantified from 5 regions of interest per organoid derived from a single well, with each well representing an independent biological replicate corresponding to mBEDOs derived from a single mouse. We observed a 9‐fold increase in lysosomal abundance in aged compared to young (Figure 1G). Staining for total mitochondria (MitoTracker Green FM, membrane potential–independent) and intact mitochondria (MitoTracker Orange CMTMRos, membrane potential–dependent) revealed a 5‐fold increase in total mitochondria and a 4‐fold increase in intact mitochondria in aged relative to young (Figure 1H). Previous reports show accumulation of mitochondria in aged cells can be attributed to impaired mitophagy (Miwa et al. 2022). Consistent with these findings, DHE staining demonstrated a 4.6‐fold increase in ROS levels in aged compared to young (Figure 1I).

Consequences of chronic oxidative stress include accumulation of DNA damage and cellular senescence, well‐established hallmarks of aging observed in the bladder urothelium (Kasturi et al. 2026; Joshi et al. 2024; Maldonado et al. 2023; Schumacher et al. 2021). Using γH2AX, a marker of DNA double‐strand breaks, we assessed the level of DNA damage in mBEDOs. Significantly higher numbers of γH2AX^+^ nuclei were observed in aged compared to young (Figure 1J). As DNA damage and oxidative stress can also activate the endoplasmic reticulum (ER)‐resident protein stimulator of interferon genes (STING), a driver of innate immune signaling and senescence (Ishikawa and Barber 2008), we assessed its expression in mBEDOs. Aged mBEDOs displayed significantly higher levels of total and phosphorylated STING compared to young (Figure 1K,L), consistent with the role of STING activation in promoting senescence during aging (Zheng et al. 2023; Li and Chen 2018). We further evaluated senescence‐associated β‐galactosidase (SA‐β‐Gal), which accumulates in senescent cells and correlates with increased lysosomal content (Debacq‐Chainiaux et al. 2009; Lee et al. 2006). We found a 2.5‐fold increase in intracellular levels of SA‐β‐Gal in aged compared to the young (Figure 1M), consistent with increased senescence reported in the aged urothelium (Joshi et al. 2024).

In aging bladder, ROS accumulates in part due to a blunted antioxidative response mediated by Nrf2 (nuclear factor erythroid 2–related factor 2) and its downstream targets (Joshi et al. 2024). Consistent with this, *Nrf2* expression was reduced by 44% in aged compared to young mBEDOs (Figure 1N), while *Nqo1* expression was increased by 92% (Figure 1O). No significant changes were observed in *Gclc* or *Hmox1* transcript levels (Figure 1P,Q), suggesting incomplete or dysregulated activation of the canonical Nrf2 antioxidant program. Together, these expression patterns indicate that aged mBEDOs retain the non‐responsive antioxidative state characteristic of the aged urothelium in vivo.

Overall, our findings demonstrate that mBEDOs capture several key molecular and structural hallmarks of urothelial aging, establishing them as a robust and scalable ex vivo model for studying epithelial aging.

### 3.2 mBEDO Platform Demonstrates Practical Utility for UPEC Infection and Macrophage Interaction Studies

Uropathogenic *E. coli* is the most common bacterial infection that accounts for 80%–90% of UTI cases in the world (Whelan et al. 2023) with increasing frequency and severity with age. We previously demonstrated that aged female mice exhibit elevated susceptibility to rUTIs, which is linked to altered urothelial barrier function, inflammation, and immune dysregulation (Joshi et al. 2024). We examined infection using 2D monolayers that maximized direct bacterial contact (Figure 2A). Monolayers were exposed to GFP‐tagged UTI89 for 1‐ or 3‐h, followed by treatment with gentamicin to eliminate extracellular bacteria and ensure that observed infections would reflect the internalized bacteria. Confocal microscopy revealed localization of UTI89 (green) with UpkIIIA^+^ cells (orange) in both young and aged 2D monolayers (Figure 2B). However, significantly higher MFI was observed in aged compared to young at 3 hpi, showing increased bacterial accumulation over time (Figure 2C). Bacterial load quantification via colony forming units (CFU) demonstrated significantly higher intracellular bacterial population in aged mBEDOs at 3 hpi (Figure 2D).

Given the complex immune landscape in the bladder, we sought to examine epithelial–immune cell interactions using the mBEDO platform. Macrophages, known to have primary functions in immune homeostasis, decline with age (Wang, et al. 2022). Using transwell inserts, we prepared separate co‐cultures of young and aged 2D monolayers with bone marrow–derived macrophages (BMDMs) from young and aged mice, respectively (Figure 2E). This system enables interaction between urothelial cells (Ck5^+^, green) on the apical compartment with the macrophages (F4/80^+^, red) on the basolateral compartment. Confocal imaging revealed co‐localization of macrophages with urothelial cells (Figure 2F). These interactions were further demonstrated on the apical and basolateral sides of the transwell inserts (Figure 2G). Together, we demonstrated the utility of this platform for studying infection and epithelial–immune cell dynamics.

### 3.3 Young and Aged mBEDOs Are Characterized by Significant and Distinct Metabolic Signatures

**FIGURE 3:**
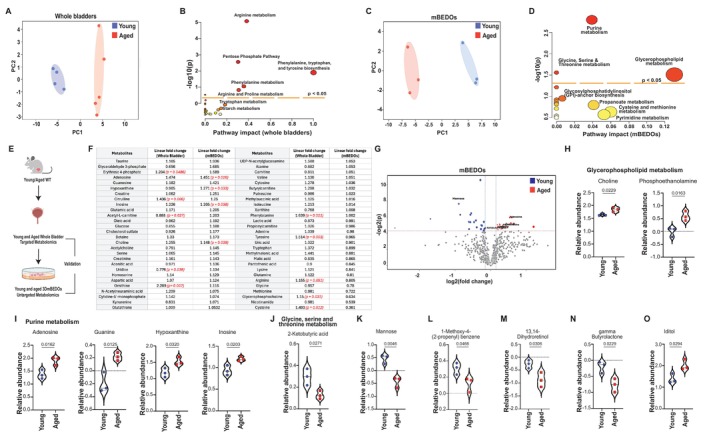
Young and aged mBEDOs are characterized by significant and distinct metabolic signatures. (A) PCA plot shows distinct clustering of metabolites from young and aged female whole bladders. (B) Pathway analysis of significantly represented metabolic pathways in the whole bladder metabolomics. Circle size correlates to the number of metabolites representing a pathway. (C) PCA plot shows distinct separation of metabolites from young and aged mBEDOs. (D) Pathway analysis of significantly represented metabolic pathways in the mBEDO metabolomics. Circle size correlates to the number of metabolites representing a pathway. (E) Validation schematic of mBEDOs using whole bladders. (F) Table shows validated metabolites organized in decreasing order of linear fold change in mBEDOs. *p* values (red) represent significantly altered metabolites in young vs. aged groups. (G) Volcano plot of metabolites differentially and significantly (*p* < 0.05) altered in mBEDOs. Relative abundance is high (red) or low (blue). (H–J) Relative abundances of metabolites from glycerophospholipid pathway, purine metabolism, and glycine, serine, threonine metabolism. (K–O) Relative abundance of Mannose, 1‐Methoxy‐4‐(2‐propenyl) benzene, 13,14‐Dihydroretinol, gamma Butyrolactone, and Iditol in young and aged mBEDOs. All Data presented as mean ± SEM, *n* = 3, *p* values by two‐tailed unpaired *t*‐test.

Metabolic dysregulation is a hallmark of aging (Zhang et al. 2021). We therefore asked whether the mBEDO platform could capture metabolic signatures associated with urothelial aging. We first performed metabolomics on whole bladders from young and aged female mice. Principal component analysis (PCA) revealed clear separation between young and aged samples, indicating distinct global metabolic profiles (Figure 3A). Pathway enrichment analysis identified several significantly dysregulated pathways including arginine and proline metabolism, phenylalanine metabolism, tryptophan, tyrosine and phenylalanine biosynthesis, and the pentose phosphate pathway (PPP) (Figure 3B). Next, we conducted metabolomics on young and aged mBEDOs to identify pathways perturbed with age in the urothelial compartment. PCA again demonstrated clear separation between mBEDO groups (Figure 3C). Pathway enrichment analysis revealed significant perturbations in glycerophospholipid metabolism, purine metabolism, and amino acid metabolism, particularly pathways involving glycine, serine, and threonine (Figure 3D).

To assess concordance between the mBEDO platform and whole bladder metabolism, we compared metabolite profiles across both datasets (Figure 3E). Fifty‐six metabolites were detected in both whole bladder and mBEDO analyses (Figure 3F). In whole bladders, metabolites including erythrose‐4‐phosphate, citrulline, ornithine, phenylalanine, tyrosine, arginine, glycerophosphocholine, and cysteine were significantly elevated in aged compared to young samples, whereas acetyl‐L‐carnitine and uridine were enriched in young bladders. Consistent with these findings, aged mBEDOs exhibited increased levels of purine‐related metabolites, including adenosine, hypoxanthine, inosine, and choline (Figure 3F).

Differential metabolite analysis in young and aged mBEDOs (*p* < 0.05) identified 41 significantly altered metabolites, with 20 decreased and 21 increased in aged organoids (Figure 3G). Among these, mannose and 2‐ketobutyric acid were reduced in aged mBEDOs, whereas adenosine, guanine, hypoxanthine, inosine, and choline were significantly elevated (Figure 3G).

We next examined changes in individual metabolites based on the pathway enrichment analysis from Figure 3D. In glycerophospholipid metabolism, choline (CH) and phosphoethanolamine (PEA) were increased in aged mBEDOs (Figure 3H). Purine metabolism was similarly perturbed with significantly elevated levels of inosine, hypoxanthine, guanine, and adenosine in aged mBEDOs (Figure 3I). In contrast, 2‐ketobutyric acid, a metabolite associated with glycine, serine, and threonine metabolism, was significantly reduced with age (Figure 3J). We also identified several biologically relevant metabolites present in the KEGG database but not confidently assigned to a specific pathway in MetaboAnalyst. Mannose (Figure 3K), 1‐methoxy‐4‐(2‐propenyl) benzene (Figure 3L), dihydroretinol (Figure 3M), and gamma‐butyrolactone (Figure 3N) were significantly decreased in aged mBEDOs. Iditol was elevated in aged mBEDOs (Figure 3O), possibly reflective of increased ROS levels.

Together, these findings demonstrate that mBEDOs undergo age‐associated metabolic shifts characterized by dysregulated redox balance, altered nucleotide turnover, and shifts in carbohydrate utilization, all hallmark features of urothelial aging. These alterations are consistent with broader mitochondrial and tricarboxylic acid (TCA) cycle remodeling that accompanies aging and intersect with immunometabolic pathways (Borkum 2023; Kurhaluk 2024).

### 3.4 mBEDO Platform Is Suitable for Generating Genetic Knockout Organoid and Performing Metabolomics Analysis

Age‐related TCA cycle changes include induction of *Irg1*, which catalyzes the production of the immunometabolite, itaconate from aconitate (Wu et al. 2022) (Figure 2A). Whole‐bladder RNA‐seq data from young and aged mice (Ligon et al. 2020) revealed *Irg1* is significantly upregulated with age, suggesting a contribution to age‐related metabolic shifts (Figure S1A), implicating *Irg1* in mediating metabolic shifts in bladder aging.

**FIGURE 4:**
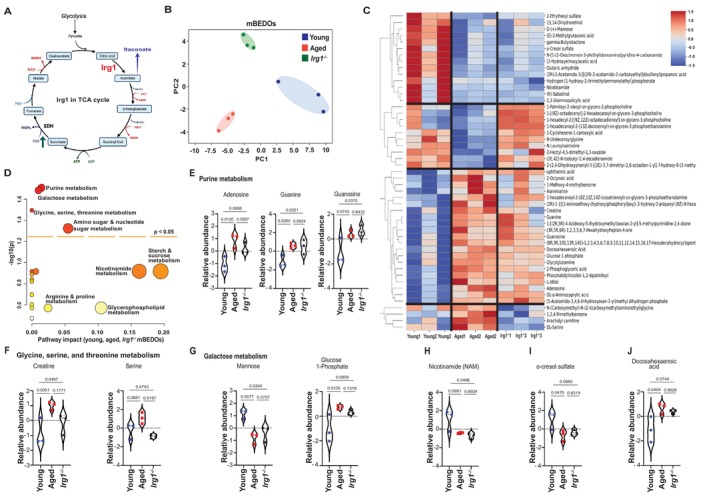
mBEDO platform is suitable for generating genetic knockout organoid and performing metabolomics analysis. (A) *Irg1* in TCA cycle. (B) PCA plot shows distinct separation of metabolites from mBEDO groups. (C) Heatmap shows differentially and significantly (*p* < 0.05) altered metabolites from mBEDO groups. (D) Pathway analysis of significantly represented metabolic pathways in mBEDO metabolomics. Circle size correlates to the number of metabolites representing a pathway. (E–G) Relative abundance of metabolites from purine metabolism, glycine, serine, threonine metabolism, and galactose metabolism. (H–J) Relative abundance of Nicotinamide, o‐cresol sulfate, Docosahexaenoic acid. All data presented as mean ± SEM, *n* = 3, *p* value by One‐way ANOVA.

We generated *Irg1* ^*−/−*^ mBEDOs (Figure S1B) and performed comparative metabolomic profiling to assess *Irg1* loss and its metabolic effect on urothelial aging. PCA plots of profiles of *Irg1* ^*−/−*^ compared to young and aged mBEDOs revealed three distinct clusters (Figure 4B) with 21 metabolites significantly increased and 15 metabolites decreased in aged and *Irg1* ^*−/−*^ compared to young WT mBEDOs (Figure 4C). We also observed 10 metabolites significantly higher in young and *Irg1* ^*−/−*^ but lower in aged mBEDOs. Further, we found 4 significantly low abundant metabolites in both young and *Irg1* ^*−/−*^ but increased in aged mBEDOs (Figure 4C).

Next, we conducted pathway analysis and identified enrichment in purine, galactose, glycine–serine–threonine, and nucleotide sugar metabolism pathways (Figure 4D). Closer inspection of individual metabolites shows specific similarities between aged and *Irg1* ^*−/−*^. In purine metabolism, adenosine was higher in aged whereas guanine was significantly elevated in both aged and *Irg1* ^*−/−*^, and guanosine was higher in *Irg1* ^*−/−*^ compared to the young (Figure 4E). In amino acid metabolism, creatine was increased in aged but not in *Irg1* ^*−/−*^ compared to the young, suggesting partial divergence in metabolic signatures whereas serine was increased in aged compared to the *Irg1* ^*−/−*^ (Figure 4F). In galactose metabolism, we found lower mannose abundance in both aged and *Irg1* ^*−/−*^ (Figure 4G), whereas glucose‐1‐phosphate was higher in aged compared to young (Figure 4G). We also examined biologically relevant metabolites that were significantly altered but not confidently assigned to a single pathway and found nicotinamide was reduced in *Irg1* ^*−/−*^ (Figure 4H), o‐cresol sulfate was reduced in aged (Figure 4I), and docosahexaenoic acid was increased in aged relative to young (Figure 4J).

Together, these findings demonstrate that *Irg1* deficiency in young mBEDOs partially phenocopies age‐related metabolic reprogramming, particularly within purine and galactose metabolism. Given that *Irg1* encodes the enzyme responsible for itaconate production, a metabolite that modulates cellular redox balance, these data suggest that disruption of *Irg1*–itaconate signaling, even in a young context, contributes to urothelial metabolic dysfunction and potentially impacts age‐associated decline.

### 3.5 mBEDOs Provide a Platform for High Throughput Supplementation Assays

Although several pharmacological inventions have been proposed to alleviate the negative impacts of aging (Guarente et al. 2024), a scalable model to evaluate pharmacological therapies has not been explored. As our metabolomics screen identified NAM and d‐mannose as being depleted in aged bladders, we set out to examine their potential effects in aging. NAM and its precursors are widely implicated in promoting health span and lifespan extension across multiple model systems (Imai and Guarente 2014; Anderson et al. 2003), whereas d‐mannose is reported to provide senotherapeutic effects in the aged female bladder in vivo (Joshi et al. 2024).

**FIGURE 5:**
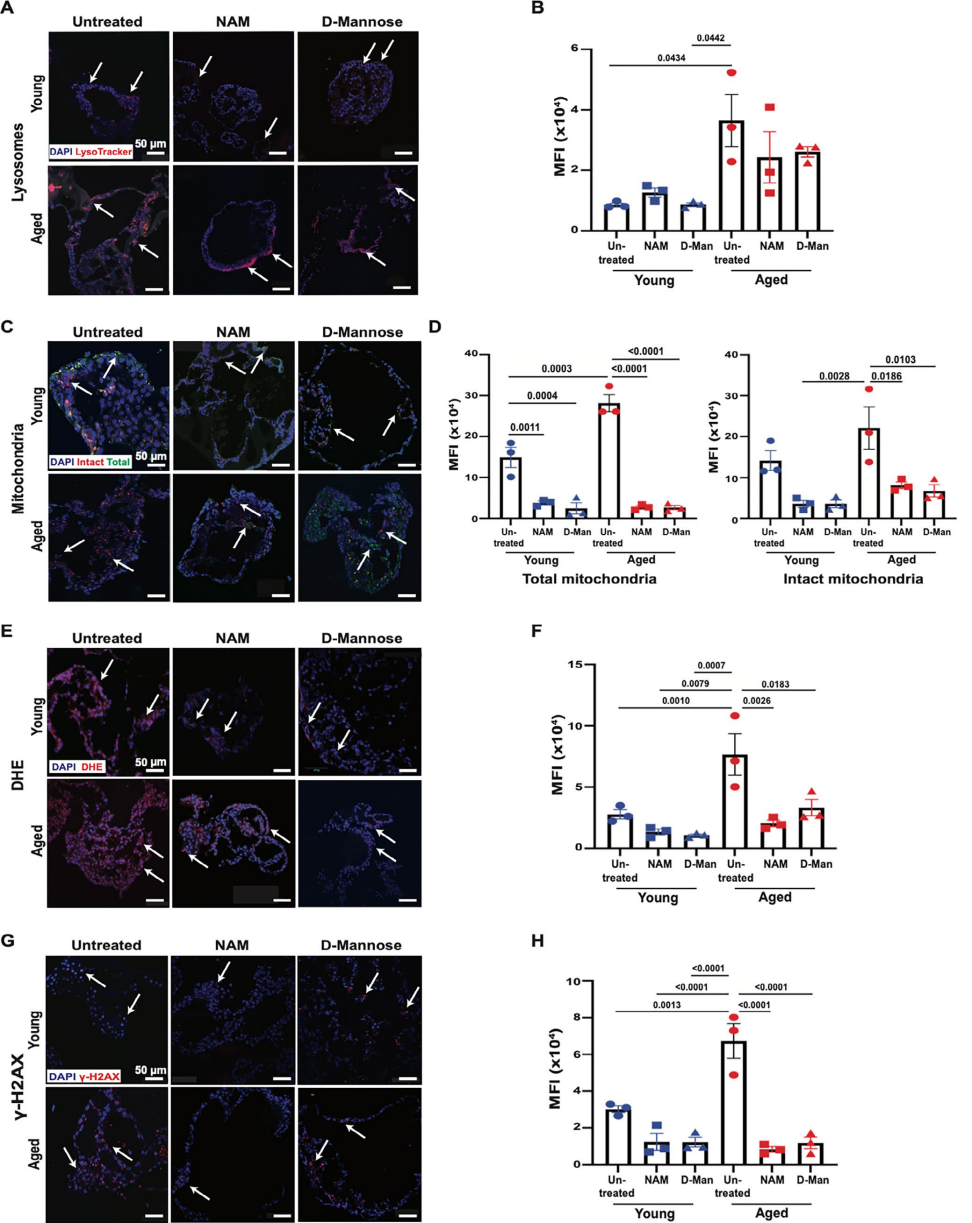
mBEDOs provide a platform for high throughput supplementation assays. (A, B) Images and quantitation of lysosome staining. (C, D) Images and quantitation of mitochondrial staining. (E, F) Images and quantitation of DHE staining. (G, H) Images and quantitation γ‐H2AX (red) (all data presented as mean ± SEM, *n* = 3, *p* value by 2‐way ANOVA, Tukey's multiple comparisons test).

We generated differentiated mBEDOs from young and aged female whole bladders and evaluated their responses to 1 mM NAM or 17.5 mM d‐mannose supplementation for 24 h. Metabolite concentrations were determined using previous reports showing no cytotoxicity at similar concentrations in stem cell‐based and organoid cultures (Guo et al. 2018; Meng et al. 2018; Yangzom et al. 2023). Untreated and treated mBEDOs were analyzed for lysosomal and mitochondrial alterations, ROS, and DNA damage. Supplementations in aged mBEDOs generally showed reduction in lysosomal abundance compared to the untreated group; however, we did not find any statistical significance (Figure 5A,B).

Other studies showed that d‐mannose improves mitochondrial membrane potential, while NAM improves the quality of mitochondria via mitophagy (Deng et al. 2022; Jang et al. 2012). We found total mitochondria in young mBEDOs were significantly reduced both in NAM (*p* = 0.0011) and d‐mannose‐treated groups when compared to untreated (Figure 5C,D). In addition, the aged mBEDOs showed similar significant responses in both treatment groups (NAM and d‐mannose, *p* < 0.0001) (Figure 5C,D). Intact mitochondria populations in young untreated and treated mBEDOs did not show any significant differences (Figure 5C,D). However, aged mBEDOs showed 4‐ and 3‐fold significant reductions in NAM and d‐mannose‐treated groups, respectively (Figure 5C,D).

Using DHE staining, we assessed the ROS levels after mBEDOs were treated with NAM and d‐mannose. We did not observe any significant change in the young mBEDOs in both treated groups when compared to untreated (Figure 5E,F). However, aged mBEDOs showed significantly reduced ROS levels in both treated groups (Figure 5E,F). This reduction in oxidative stress is consistent with the observed reduction in mitochondrial abundance in both treatment groups in aged mBEDOs.

Young mBEDOs did not show any significant alterations in the levels of DNA damage in both treated groups when compared to untreated (Figure 5G,H). However, both NAM‐ and d‐mannose‐treated aged mBEDOs showed significantly reduced DNA damage (Figure 5G,H), suggesting improvement in nuclear integrity.

Finally, we assessed whether the concentration levels of NAM or d‐mannose elicited cytotoxic effects. Neither NAM nor d‐mannose treatment altered lysosomal abundance, intact mitochondrial levels, ROS, or DNA damage relative to untreated young mBEDOs, indicating no overt toxicity. We noted that young mBEDOs treated with d‐mannose had significantly lower abundance of lysosomes compared to the aged untreated group shown in Figure 5B. In addition, both NAM‐ and d‐mannose–treated young mBEDOs showed reduced oxidative stress (Figure 5F) and markedly lower levels of DNA damage (*p* < 0.0001) relative to aged untreated mBEDOs (Figure 5H). Together, these findings suggest that metabolic interventions targeting redox homeostasis and carbohydrate metabolism can modulate aging‐associated stress phenotypes, underscoring the utility of mBEDOs as a scalable platform for testing metabolic strategies.

## 4 Discussion

Organoids have contributed to the advancement of lower urinary tract biology from urothelial responses during UPEC infection to recapitulation of bladder cancer (Mullenders et al. 2019). Our study demonstrates a practical, scalable, and reproducible platform for generating organoids from mouse whole bladders to study urothelial aging. Characteristics of the in vivo mouse aged bladders are recapitulated by the aged mBEDOs, such as reduced growth, lysosomal and mitochondrial accumulation, oxidative stress, DNA damage, insufficient antioxidative response, and senescence. Our 3‐stage mBEDO protocol, consisting of proliferation, cell expansion, and cell differentiation, demonstrates the production of differentiated, multilayer, and central lumen‐containing organoids for studying urothelial‐intrinsic phenotypes. Our model adds to the growing body of ex vivo organoid models (Smith et al. 2006; Mullenders et al. 2019; Kim et al. 2020; Azar et al. 2021; Torrens‐Mas et al. 2021; Sato et al. 2009; Walz et al. 2023) and permits uniquely capturing tissue‐specific aging phenotypes and metabolic remodeling, offering a translational framework for developing and testing interventions that target key hallmarks of urothelial aging.

Intestinal organoids have been used to evaluate macrophage and epithelial interactions (Recaldin et al. 2024; Múnera et al. 2023) and gastric and lung organoid studies have assessed infections caused by *Helicobacter pylori* and the respiratory syncytial virus respectively (Clevers 2016; Lawrence et al. 2024; van Dijk et al. 2024). These models are useful in understanding inflammatory and pathogen‐triggered immune responses. We leveraged the mBEDO platform for interrogating age‐related mechanistic underpinnings of both immune‐epithelial interactions and infections. Our platform demonstrates epithelial colonization and increasing bacterial burden after infecting aged mBEDOs with UPEC, consistent with previous reports on age‐associated increase in rUTIs in vivo (Ligon et al. 2023). mBEDOs' amenability to co‐culture with macrophages further amplifies applications of our platform in immune–epithelial interactions, which are often difficult to dissect in vivo due to the complexity of tissue microenvironments.

Organoids are emerging as invaluable tools for modeling metabolic signatures and exploring high‐throughput metabolite supplementation (Murphy and Sweedler 2022; Torrens‐Mas et al. 2021). In recent years, metabolomics studies have been performed in organoid cultures in the context of pancreatic, kidney, as well as bladder cancer and infectious models (Keilberg et al. 2021; Walz et al. 2023), however, the targeted use of organoids specifically to address metabolic underpinnings of aging independent of disease has not been explored. Targeted metabolomics screen in young and aged whole mouse bladders revealed significant differences between the two groups. Specifically, we noted arginine metabolism, phenylalanine, tyrosine, tryptophan, and proline metabolism, as well as the PPP were over‐represented in our data. These data were particularly interesting because PPP has been implicated for its role in driving age‐associated decline in renal function and in Alzheimer's disease (Han et al. 2024; Palmer 1999). Interestingly, PPP byproducts are used for de novo nucleotide biosynthesis which falls under purine metabolism (Lane and Fan 2015). We found several metabolites (inosine, hypoxanthine, guanine, and adenosine) increased in the aged mBEDO samples could be mapped to the purine pathway. Purine metabolism was reported to increase in aged cardiac mouse tissue (Willems et al. 2003), and a dysregulated purine metabolism contributes to the progression of LUTS in the elderly (Birder and Jackson 2021). Previous reports show aged human fibroblasts produce more inosine and adenosine than those from young donors (Ethier et al. 1989). The damage caused by ROS to DNA may lead to the accumulation of 8‐oxo‐guanine lesions at telomeres, possibly contributing to increase in guanine in aged mBEDOs (Barnes et al. 2022). Also, exposure to hypoxanthine, known to increase ROS, may exacerbate bladder aging (Birder et al. 2024).

We used whole bladder metabolites to identify conserved and overlapping metabolites in the mBEDO metabolomics data. Upon validating our metabolomic findings in mBEDOs using our whole bladder metabolomics, we found an overlap of 56 metabolites. Metabolites were organized in order of decreasing linear fold change and showed taurine to have the highest fold delta when comparing young vs. aged mBEDOs, opening the possibility for future investigations of amino acid‐based therapies like tauroursodeoxycholic acid or TUDCA to ameliorate bladder aging‐associated ER stress and dysfunction. PEA and CH levels were increased in aged mBEDOs. PEA accumulation is linked to senescence during aging, while elevated CH has been associated with overactive bladder symptoms, suggesting a link between lipid metabolism and functional decline in aging bladder (Tighanimine et al. 2024). Identification of metabolites in mBEDOs delineates epithelial‐specific metabolic shifts with age and lays the foundation to systematically test targeted interventions that ameliorate metabolic changes with age.

A strength of the mBEDO platform is its adaptability for modeling genetic perturbations. We successfully generated mBEDOs from whole bladders of female *Irg1* ^*−/−*^ mice. While *Irg1* has been extensively studied in myeloid cells, its role in the epithelium was unexplored. Loss of *Irg1* resulted in metabolic profiles that resemble aged mBEDOs, particularly in purine and galactose metabolism. Notably, 35 out of 49 significantly altered metabolites in our three‐way comparison followed a similar trend in aged and *Irg1* ^*−/−*^ mBEDOs, including increases in adenosine, guanine, and guanosine. This is consistent with reports of enhanced purine metabolism in *Irg1*‐deficient macrophages (Harber et al. 2024), raising the possibility that loss of *Irg1* drives aging‐associated metabolic shifts in bladder epithelial cells. Our data align with other studies that have shown itaconate is a key metabolite that offers a gero‐protective role in inflammation and bone loss (Wang, Li, et al. 2022). Interestingly, metabolites linked to amino acid metabolism, such as serine, did not differ between *Irg1* ^*−/−*^ and young mBEDOs, suggesting that TCA cycle dysregulation in this context may selectively impact certain metabolic branches. In contrast, d‐mannose, a galactose pathway metabolite and potential senotherapeutic, as well as ni were reduced in both aged and *Irg1* ^*−/−*^ mBEDOs. Together, these results show *Irg1*'s contribution to metabolic homeostasis in the urothelium and highlight mBEDOs as a powerful platform to study the intersection of metabolism, aging, and gene function in a tissue‐specific context.

Excitingly, supplementation with two age‐dysregulated metabolites, NAM and d‐mannose, restored redox balance and reduced DNA damage in aged mBEDOs. d‐mannose was shown by our group to exert gero‐protective effects in vivo in the aged bladder epithelium (Joshi et al. 2024). NAM studied for its role in lifespan extension and cellular resilience, largely due to its role as a precursor to nicotinamide adenine dinucleotide, a key cofactor in redox reactions and mitochondrial function (Anderson et al. 2003). We observed that treatment with either NAM or d‐mannose led to a marked reduction in ROS and restoration of mitochondrial homeostasis. Reduction in intact mitochondria in aged mBEDOs may indicate improved mitochondrial function and adaptive response to energy demands (Srivastava 2017). Previous studies show reduction in mitochondrial abundance offers certain advantages such as improving metabolic flexibility and extending lifespan (Srivastava 2017). Both interventions also significantly reduced γH2AX levels, indicating a reduction in DNA damage in treated mBEDOs, consistent with prior work showing that NAM and its related precursors, such as nicotinamide mononucleotide (NMN), can reduce ROS and enhance DNA repair capacity in aging (John et al. 2012; Imai and Guarente 2014). Several studies have shown NMN and nicotinamide riboside, which are both precursors of the same pathway, help enhance DNA repair (Qiu et al. 2024). Similarly, d‐mannose reduces oxidative stress in other inflammatory conditions, including a murine model of ulcerative colitis (Lu et al. 2024), and reduces osteoarthritis progression (Zhou et al. 2021).

In summary, we have established mBEDOs demonstrate key features of bladder aging, enable the study of epithelial–immune and host–pathogen interactions, and serve as a versatile platform for dissecting metabolic alterations and testing therapeutic interventions to restore epithelial homeostasis. Although our model does not yet incorporate the full complexity of bladder architecture, it offers a tractable and reproducible platform for studying urothelial aging. Future directions include extending this work to human bladder organoids derived from postmenopausal donors to reflect clinical relevance.

## Author Contributions

A.R.P., A.M.S., and I.U.M. conceived the experimental plan and wrote the manuscript; A.R.P. performed the majority of experiments and was assisted by A.M.S., M.D., D'.J.L., and A.S.S. Metabolomics and preliminary data analysis were performed by V.P. and N.P. S.J.B. analyzed the metabolomics data, and all authors approved the final draft.

## Funding

This work was supported by the National Institutes of Health (Grants R56 AG084691‐01A1, R01CA282282, and U01CA111302) and the Cancer Prevention and Research Institute of Texas (Grants RP210227 and RP210027).

## Conflicts of Interest

I.U.M. serves on the scientific advisory board of Seed Health. The remaining authors declare no competing interests.

## Supporting information


**Figure S1:** (A) RNA sequencing analysis of *Irg1* expression in young versus aged whole mouse bladders show increase of *Irg1*expression in aged whole bladders. (B) Suitability of the mBEDO model to derive genetic knockout organoids.
**Table S1:** Materials and Reagents.
**Table S2:** qRT‐PCR Primer sequences.

## Data Availability

Metabolomics data reported in this study are available on Metabolomics Workbench at https://www.metabolomicsworkbench.org/, accession numbers: ST004420 and ST004419.
