# Supplementary material for: A Scalable Organoid Model of Urothelial Aging for Metabolic Interrogation, Infection Modeling, and Reversal of Age‐Associated Changes
Source: Aging Cell. 2026 Jan 23;25(2):e70391. doi: 10.1111/acel.70391 (PMC12830083; doi:10.1111/acel.70391)
Supplement: Supplementary file 1 — Figure S1: (A) RNA sequencing analysis of Irg1 expression in young versus aged whole mouse bladders show increase of Irg1expression in aged whole bladders. (B) Suitability of the mBEDO model to derive genetic knockout organoids. Table S1: Materials and Reagents. Table S2: qRT‐PCR Primer sequences. [file ACEL-25-e70391-s001.pdf]

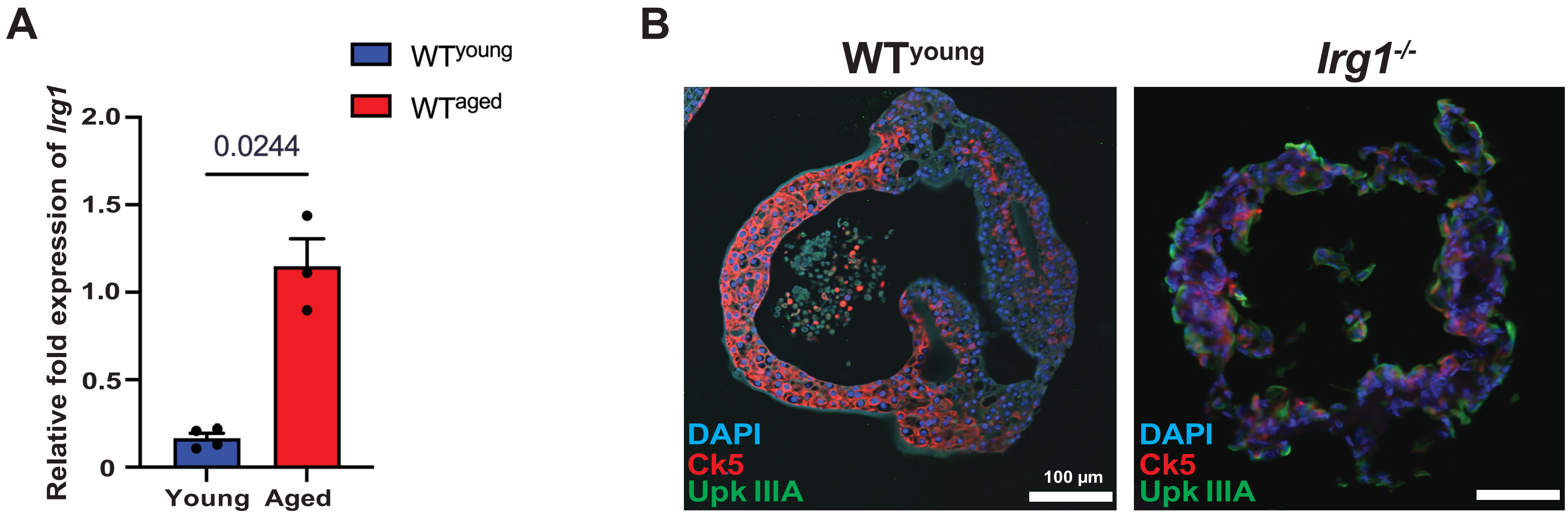

Supplementary Fig. 1. A) RNA sequencing analysis of *Irg1* expression in young vs. aged whole mouse bladders show increase of *Irg1* expression in aged whole bladders B) Suitability of the mBEDO model to derive genetic knockout organoids. Representative images of wild-type young and *Irg1*$^{-/-}$ mBEDOs showing CK5 (red) and UPKIIIA (green). DAPI (blue) stains nuclei, scale bar = 100 µm.

Supplemental Table 1. Materials and Reagents.

Reagent	Catalog Number
mBEDO generation	
1X PBS	Gibco Cat. No. 10010-031
Collagenase II	Gibco Cat. No. 17101015
Y-27632 (Rock inhibitor)	Stem Cell Technologies Cat. No. 72302
Elastase	Sigma-Aldrich Cat. No. E7885
Advanced DMEM	Invitrogen Cat. No. 12634-034
1X TrypLE	Gibco Cat. No. 12605028
100X GlutaMAX	Gibco Cat No. 350561
Matrigel	Corning Cat No. 356231
6-well suspension plate	Greiner Cat. No. 657185
50X B27	Gibco Cat. No. 17504-044
N-Acetyl-L-Cysteine	Sigma-Aldrich Cat. No. A9165-5G
NAM	Sigma-Aldrich Cat. No. N0636-100G
mouse EGF	Life Technologies Cat. No. PMG8041
A-83-01	Tocris Cat. No. 2939
Pen Strep	Invitrogen Cat. No. 15140-122
mouse IL-6	Cell Signaling Technologies Cat No. 5216
FBS	Gibco Cat No. A5670801
mouse FGF-7	PeproTech Cat. No. 450-60
mouse FGF-10	PeproTech Cat. No. 450-61
Recovery Cell Culture Freezing Medium	Gibco Cat No. 12648010
Antibodies	
anti-Upk IIIA	Fitzgerald Cat No. 10R-U103a
anti-Ck5	Abcam Cat No. ab53121
anti-γH2AX	Cell Signaling Technology Cat No. 9718S
anti-SA-β-Gal	Cell Signaling Technology Cat No. 27198S
anti-STING	Cell Signaling Technology Cat. No. 13647T
anti-pSTING	Cell Signaling Technology Cat. No. 50907T
anti-F4/80	Abcam Cat No. ab6640
Alexa Fluor Goat anti-Mouse 488	Invitrogen Cat No. A11029
Alexa Fluor Goat anti-Rabbit 594	Invitrogen Cat No. A11037
Alexa Fluor Goat anti-Rabbit 488	Invitrogen Cat No. A11034

Staining Reagents	
OCT	Sakura Tissue Tek OCT Cat. No. 4583
Bovine serum albumin (BSA)	Sigma-Aldrich Cat. No. A7030
13mm diameter x 0.8 mm depth silicone isolators	Grace Bio-Labs Cat. No. JTR13R-1.0
Paraformaldehyde (PFA)	Thermo Scientific Cat. No. 047317.9M
Hematoxylin	Epredia Cat. No. 7211
Eosin	Epredia Cat. No. 71204
Permount mounting medium	Fisher Chemical Cat. No. SP15-100
Lysotracker dye	Invitrogen Cat. No. L7528
MitoTracker™ Orange CMTMRos	Invitrogen Cat. No. M7510
MitoTracker™ Green FM	Invitrogen Cat. No. M7514
Dihydroethidium (DHE) dye	Thermo Fisher Scientific Cat#D11347
DAPI	Invitrogen Cat. No. P36931
qRT-PCR	
TRIzol™ Reagent	Thermo Fisher Scientific, 15596018, USA
chloroform	Sigma-Aldrich Cat. No. 288306
DNase I treatment	Thermo Fisher Scientific, 18068-015, USA
SuperScript™ II Reverse Transcriptase	Thermo Fisher Scientific, 18064-014, USA
RNase-free water	Invitrogen Cat. No. 46-2224
SsoAdvanced Universal SYBR™ Green Supermix	Bio-Rad, 1725274, USA
UPEC infection	
Gentamicin	Gibco Cat. No. 15750-060
BMDM co-culture with organoids	
1XRBC lysis buffer	420301, BioLegend
M-CSF	315-02, Peprotech

Supplemental Table 2. qRT-PCR Primer sequences.

Gene	***Direction***	***Sequence***
Mouse Nrf2	*Forward*	*AACAGAACGGCCCTAAAGCA*
Mouse Nrf2	*Reverse*	*GGGGTTCACGCATAGGAGCA*
Mouse Nqo1	*Forward*	*AGGATGGGAGGTACTCGAATC*
Mouse Nqo1	*Reverse*	*AGGCGTCCTTCCTTATATGCTTA*
Mouse Gclc	*Forward*	*GGGGTGACGAGGTGGAGTA*
Mouse Gclc	*Reverse*	*GTTGGGGTTTGTCCTCTCCC*
Mouse Hmox1	*Forward*	*AAGCCGAGAATGCTGAGTTCA*
Mouse Hmox1	*Reverse*	*GCCGTGTAGATATGGTACAAGGA*
